# Supplementary material for: Altered microRNA Transcriptome in Cultured Human Liver Cells upon Infection with Ebola Virus
Source: Int J Mol Sci. 2021 Apr 6;22(7):3792. doi: 10.3390/ijms22073792 (PMC8038836; doi:10.3390/ijms22073792)
Supplement: Supplementary file 1 [file ijms-22-03792-s001.zip › Supplementary_File/D_ KEGG_Analysis_Results/16-30nt_pathway_Reston-96h-Huh7_vs_Control-96h-Huh7_down.mature_mirna_targets/hsa_GeneRatioDotPlot.pdf]

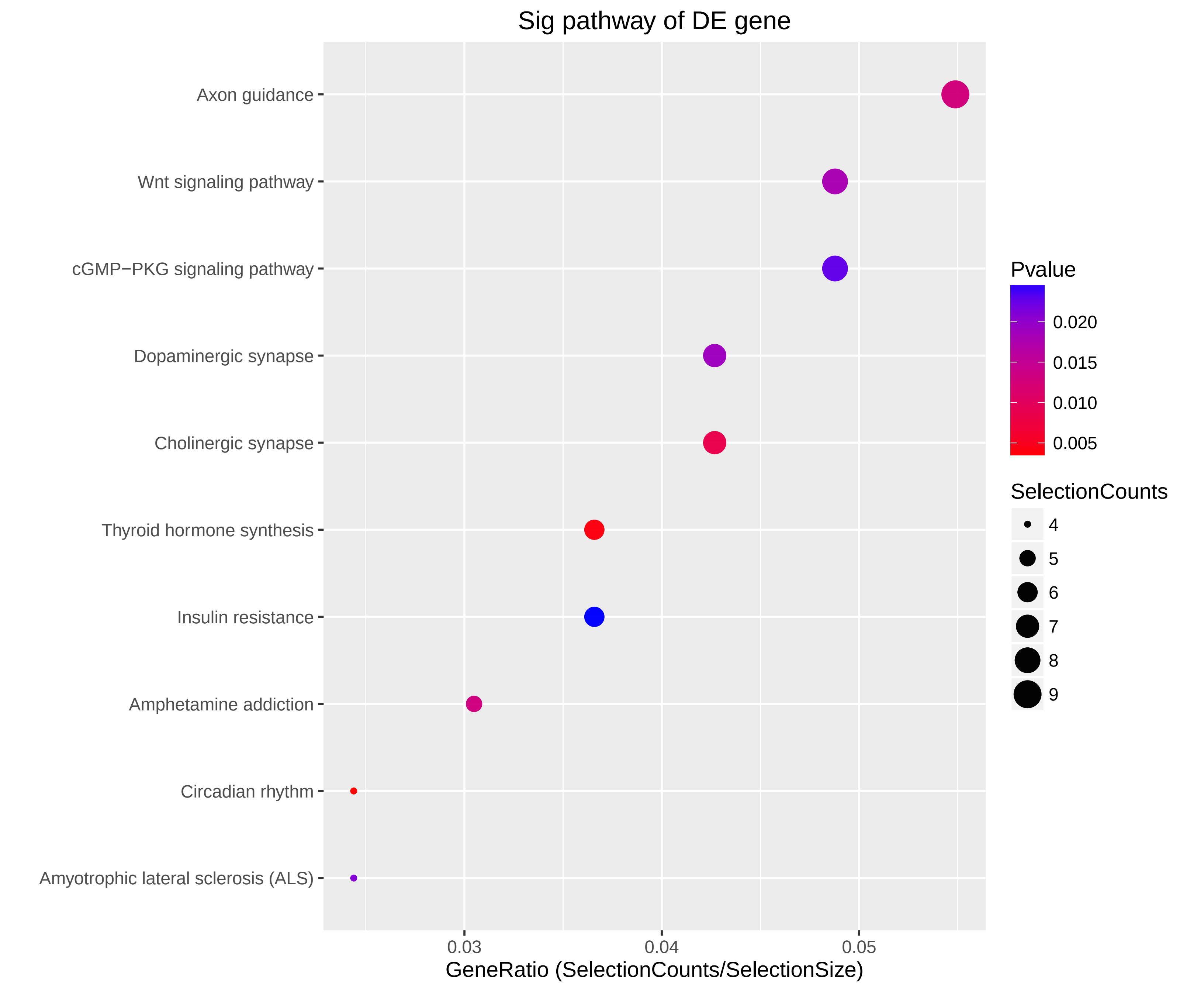
Sig pathway of DE gene
Axon guidance
Wnt signaling pathway
cGMP−PKG signaling pathway
Dopaminergic synapse
Cholinergic synapse
Thyroid hormone synthesis
Insulin resistance
Amphetamine addiction
Circadian rhythm
Amyotrophic lateral sclerosis (ALS)
0.03
0.04
0.05
GeneRatio (SelectionCounts/SelectionSize)
Pvalue
0.020
0.015
0.010
0.005
SelectionCounts
4
5
6
7
8
9
